# Detection of invasive *Aedes vittatus* mosquitoes in Jamaica: molecular identification and surveillance implications

**DOI:** 10.1186/s13071-025-07066-6

**Published:** 2025-11-18

**Authors:** Simmoy A. A. Noble, Reneé L. M. N. Ali, Cameil F. Wilson-Clarke, Nadia K. Khouri, Douglas E. Norris, Simone L. Sandiford

**Affiliations:** 1 Department of Microbiology, Faculty of Medical Sciences, The University of the West Indies Mona, Kingston, Jamaica; 2 The W. Harry Feinstone Department of Molecular Microbiology and Immunology, Johns Hopkins Malaria Research Institute, Johns Hopkins Bloomberg School of Public Health, Baltimore, MD USA; 3 Department of Basic Medical Sciences, Pharmacology and Pharmacy Section, Faculty of Medical Sciences, The University of the West Indies, Mona, Kingston, Jamaica; 4 Mosquito Control and Research Unit, The University of the West Indies, Mona, Kingston, Jamaica

**Keywords:** *Aedes vittatus*, Invasive species, Jamaica

## Abstract

**Background:**

*Aedes vittatus*, an emerging invasive mosquito of significant public health concern has slowly made its way onto the global radar. With a known geographical range in Africa and Asia, where it is a competent vector for several arboviruses, this mosquito has now been reported in the Americas. As the spread of this mosquito has been partly linked to transcontinental trade and travel, Jamaica, the largest English-speaking country in the Caribbean, which serves as a central hub for trade and transport throughout the region, has been on alert since its identification in neighboring Dominican Republic and Cuba.

**Method:**

BG sentinel traps baited with dry ice and a Prokopack aspirator were used to collect adult mosquitoes whereas disposable plastic pipettes were utilized for the collection of immature stages. Larvae were reared to adults, and all mosquitoes were identified using taxonomic keys. Using a genome skimming approach, the mitochondrial genome from two specimens was sequenced and a section of the *cytochrome c oxidase subunit I* gene was extracted from each mitochondrial genome and used for phylogenetic analysis.

**Results:**

Through ongoing surveillance efforts from January 2023 to October 2024, we report the detection of *Ae*. *vittatus* across six locations in four parishes in Jamaica. Both larvae and adults were collected from rural and urban areas in the country. Additionally, we present the first complete annotated mitochondrial genomes from two specimens of this invasive mosquito species. Phylogenetic analysis using the *cytochrome c oxidase subunit I* gene extracted from the derived mitochondrial genomes of Jamaican *Ae*. *vittatus* and available sequences from the GenBank database revealed clustering with specimens from Cuba, Nepal, and India.

**Conclusions:**

This study is the first confirmed report of *Ae*. *vittatus* in Jamaica. Furthermore, it highlights the benefits of routine surveillance and the power of molecular approaches to identify invasive species and their potential origins.

**Supplementary Information:**

The online version contains supplementary material available at 10.1186/s13071-025-07066-6.

## Background

Reports of invasive arboviral vectors and mosquito-borne diseases of public health and veterinary concern have steadily increased across the globe [1]. Human-mediated activities, including trade and tourism, are among the primary pathways through which invasive species are introduced into new habitats [2]. In our interconnected society, the seamless mobility of people and objects facilitated by advancement and growth in transportation have profound implications for the introduction of species to new habitats and the transmission of infectious diseases [3]. Additionally, ecosystems may be altered by invasive species and resource competition may result in the displacement of existing species [4].

Jamaica’s position as a prominent tourist hotspot makes it vulnerable to the introduction and dissemination of re-emerging vectors and infectious agents. The country has seen a significant rise in total visitor arrivals to the island, increasing from 1,535,165 in 2021 [5] to 4,181,740 in 2023 [6]. Jamaica also functions as a transportation hub for the Caribbean region and has longstanding preferential bilateral trade agreements with countries such as Barbados, Guyana, and Trinidad and Tobago. Furthermore, since the early 2000s Jamaica has established key partnerships with Cuba [7] and the Dominican Republic [8], and increases in both exports and imports have been noted within the last 2 years.

In the last century, the Asian tiger mosquito *Aedes albopictus* has been the most successful invasive mosquito [9]. Native to Asia, this mosquito has become ubiquitous in the Caribbean and the Americas, and was reported in Jamaica in 2019 [10]. The distribution of this invasive species was facilitated primarily through human trade, where eggs were unintentionally transported on old tyres and decorative plants. Adult mosquitoes were also inadvertently carried by public and private ground transit from highly infested regions [9].

Currently, there is a new and emerging vector in the Western Hemisphere, *Ae*. *vittatus*. A member of the subgenus *Fredwardsius*, *Ae*. *vittatus* is native to Asia and parts of Africa [11, 12]. This, however, is rapidly changing in the New World, as *Ae*. *vittatus* has been reported in both the Dominican Republic [13] and Cuba [14–16]. Genetic analysis conducted on specimens from both countries using short sequence fragments of the mitochondrial *cytochrome c oxidase subunit I* (COI) gene suggests multiple introductions from Asia into the Caribbean [13, 14].

Increasingly, complete mitochondrial genomes (mitogenomes) are being utilized to provide greater insights into the evolutionary histories of mosquitoes [17]. While mitogenomes of the highly invasive *Ae. albopictus* mosquito [18–21] in addition to other invasive vectors, such as *Ae*. *vexans* [22], *Ae*. *japonicus* [23, 24], and *Ae*. *scapularis* [25], have all been reported, no such data currently exists for *Ae*. *vittatus*.

*Aedes vittatus* is known to be a competent vector for several arboviruses, including dengue [26], chikungunya [27, 28], Zika [29], yellow fever [12], and West Nile viruses [30]. Factors such as climate change, urbanization, lack of effective vector control [31], and resilient eggs [32] have allowed this invasive mosquito to thrive in new environments. Adults have been captured from forest, Savannah, and barren land [33]. The ability of *Ae*. *vittatus* to survive in a wide range of habitats speaks to its high environmental plasticity and is reflected in oviposition habitats ranging from natural to artificial containers. In Africa, *Ae*. *vittatus* primarily lay eggs in rock holes [34], and larvae have been collected from tree holes, fresh fruit husk, and puddles. However, in peridomestic habitats *Ae*. *vittatus* is predominantly found in artificial containers such as tyres, bottles, cups, and potted plants, demonstrating the urbanization of this species in Nigeria, India, and Pakistan [12, 35]. In southern India, larvae were collected from cement tanks, cement cisterns, and mud pots [36]. Similar to what has been seen with *Ae*. *albopictus*, displacement to new regions and destruction of natural habitats has forced *Ae*. *vittatus* to adapt to breeding in human-made containers [1].

Addressing the emerging threat of invasive mosquitoes like *Ae*. *vittatus* requires a comprehensive approach that combines traditional entomological approaches and molecular tools. These include routine surveillance and reference sequence data for the development of innovative vector control strategies. Despite the potential public health significance of *Ae*. *vittatus*, information on its presence and genetic characteristics remains scarce within the Caribbean. This lack of detection and genomic reference data hampers surveillance and limits the development of targeted control measures. This study aims to report the occurrence of *Ae*. *vittatus* in Jamaica and generate genomic reference data to support future surveillance and vector control efforts in the Caribbean.

## Method

### Study area

**Fig. 1:**
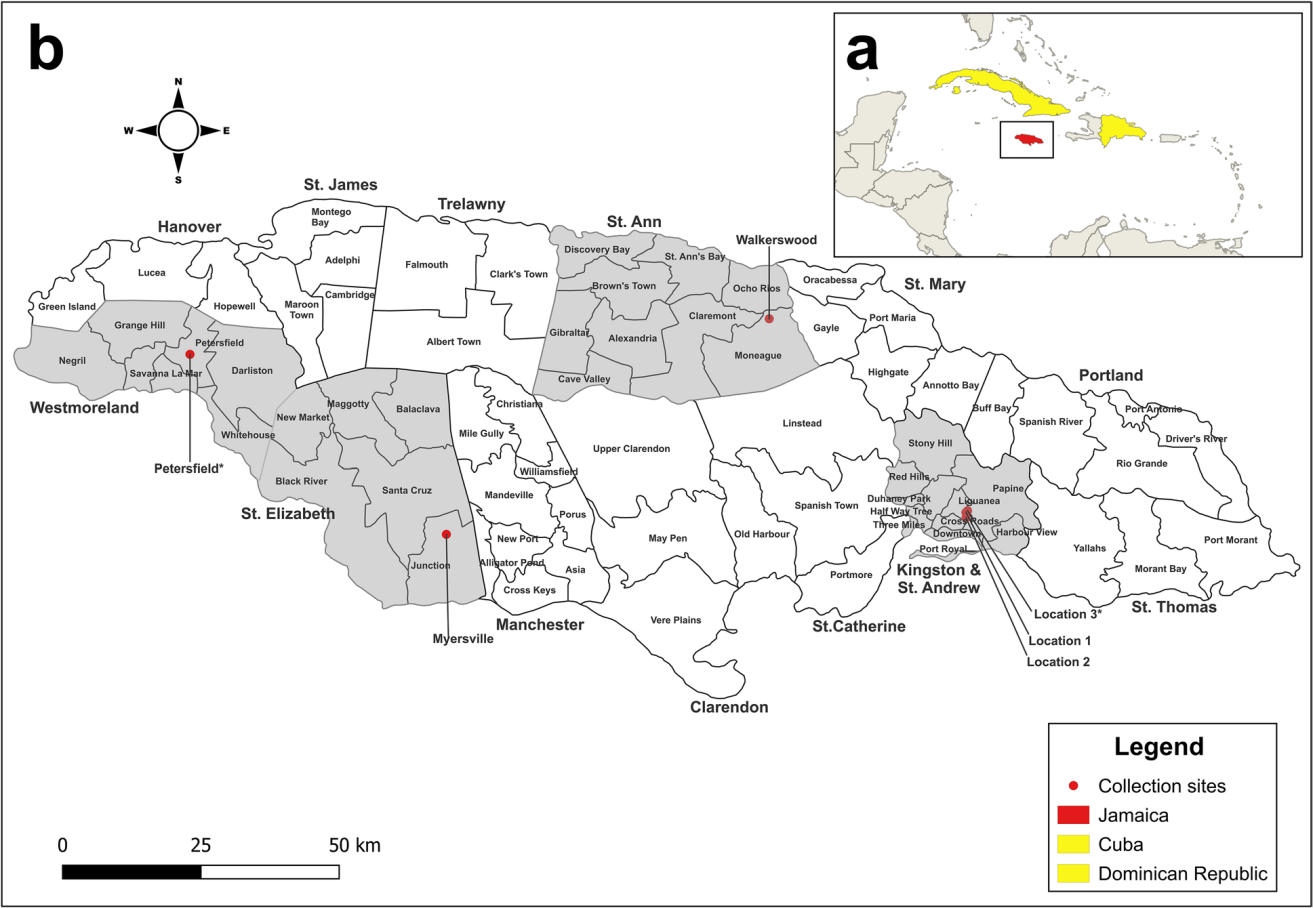
Map of Jamaica and sampled sites across the country. (**a**) the geographic location of the country in the Caribbean and (**b)** a detailed view of Jamaica and its parishes. Collection sites are represented by the red dots and the asterisk indicates specimens that were used for mitogenome analysis. Administratively, the parishes of Kingston and St. Andrew are combined however, locations 1, 2 and 3 are in the parish of St Andrew. Yellow highlights countries in the Americas that have previously reported *Ae*. *vittatus*. Map was created using QGIS 3.34.11

**Fig. 2:**
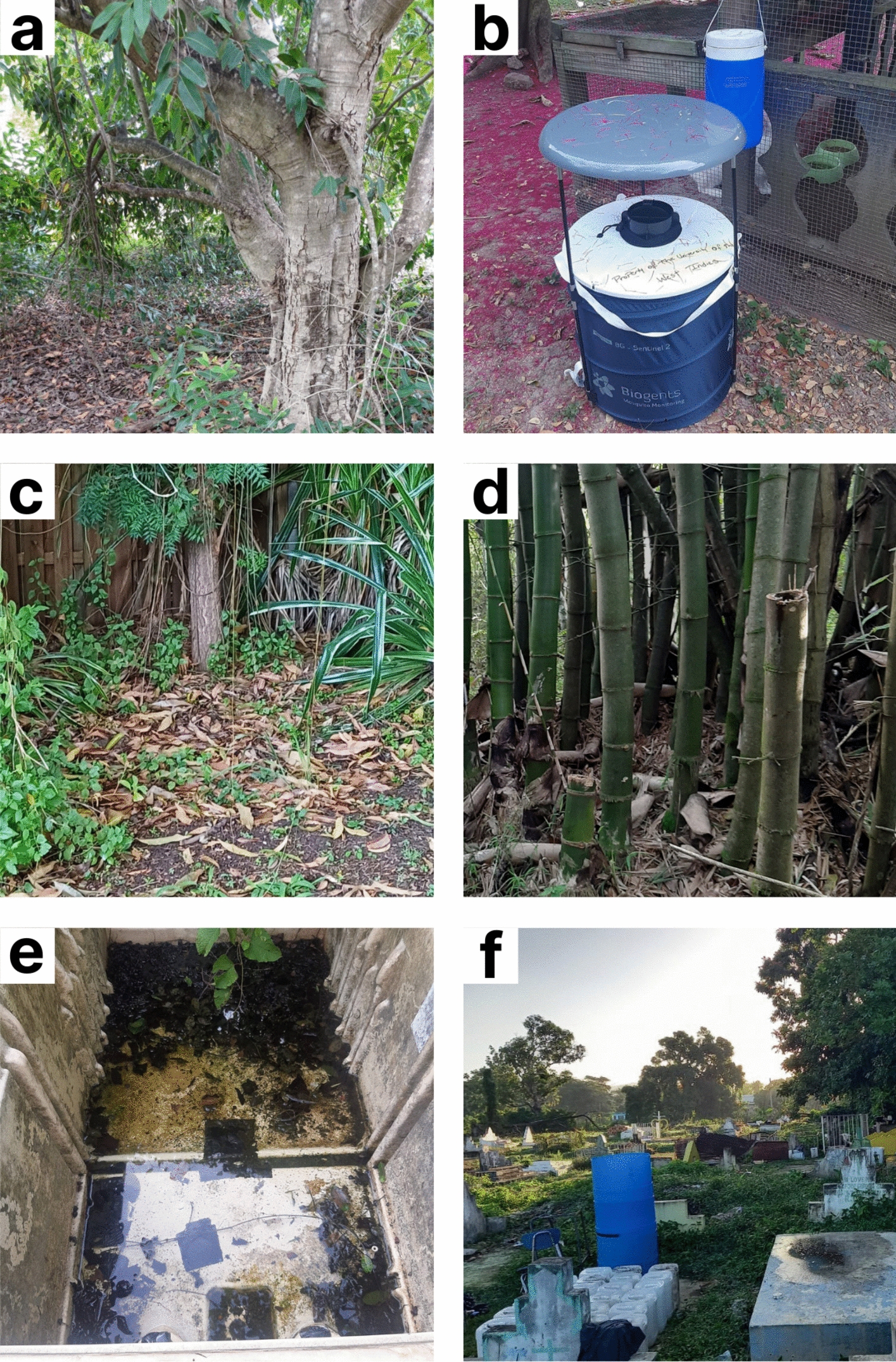
Locations where *Ae*. *vittatus* was identified. **a** Location 1 Mona, St. Andrew. **b** Location 2 Mona, St. Andrew. **c** Location 3 Mona, St. Andrew. **d** Walkerswood, St. Ann. **e** Myersville, St. Elizabeth. **f** Petersfield, Westmoreland

As part of an ongoing arboviral surveillance project, convenience sampling was utilized to collect mosquito specimens from January 2023 to October 2024. Locations in St. Andrew were in and around the university, thus facilitating ease of collections. Sampling of rural sites in St. Ann, St. Elizabeth and Westmoreland coincided with community engagement projects in those areas at that time. Mona is a peri-urban neighborhood in the southeastern parish of St. Andrew, located 8 km from the capital of Kingston (Fig. 1 and Fig S1). It is a former sugar plantation and is where The University of the West Indies and a fresh-water reservoir are situated. Location 1 (N18.007352 W-76.751662) was an area of mixed vegetation with domestic animals (horses, sheep, and chickens) present nearby (Fig. 2a). Location 2 (N17.9987 W-76.751483) was a private residential community surrounded by a heavily forested area (Fig. 2b) and was approximately 990 m from location 1. Location 3 (N18.01007 W-76.748885) was a lot with overgrown shrubs and trees with a single building adjacent to both residential and abandoned houses (Fig. 2c), and was 410 m from location 1 and 1290 m from location 2.

Walkerswood (N18.320017 W-77.08635) is a rural farming community in the parish of St. Ann in northern Jamaica (Fig. 1). The collection site was a forested region at the back of a church, characterized by bamboo clusters and small to medium trees (Fig. 2d). There was an indication of human activity at this site as many of the bamboo trees were deliberately cut down leaving the roots intact in the earth, and discarded containers were also present (Fig S2).

Myersville (N17.971833 W-77.635567) is a rural farming community in the parish of St. Elizabeth located in southwest Jamaica (Fig. 1). Two locations were surveyed, the first a bushy area, with a large human-made water catchment area which provided a habitat for frogs and birds, and the second a forested area with large, discarded refrigerators positioned in the shade. The water inside the refrigerators was clear but contained decaying plant material (Fig. 2e).

Petersfield (N18.262467 W-78.071733) is a rural sugarcane producing community in the parish of Westmoreland, is situated 41 km southwest of Montego Bay, the second major city in Jamaica (Fig. 1). The collection site was a cemetery located behind a church and adjacent to a school and a heavily forested area (Fig. 2f).

### Mosquito collection

Owing to the proximity of the St. Andrew sites to The University of the West Indies, BG sentinel traps (Biogents, Germany) without BG chemical lure were set from 14:00 to 22:00 h and baited with 2 lbs of dry ice to collect mosquitoes. At location 1 in Mona (Fig S1) the trap was placed in close proximity to human habitation, and positioned in a shaded area under a tree, surrounded by overgrown shrubs (Fig. 2a). For location 2 (Fig S1) the trap was placed under a tree beside an existing rabbit cage, approximately 7 m from a residential house, which was surrounded by a forested area (Fig. 2b). At location 3 in Mona (Fig S1) the trap was positioned in a shaded area surrounded by overgrown vegetation (Fig. 2c). Because of logistical challenges involved in conducting overnight trapping sessions at the rural locations, a Prokopack aspirator (John W. Hock, USA) was used to aspirate specimens from shrubs, during the hours of 13:00 to 15:00 h at the St. Ann (Fig. 2d) and St. Elizabeth sites (Fig. 2e), and from 11:00 to 14:00 h at the Westmoreland sites (Fig. 2f). Adult specimens were transported to the laboratory on dry ice and stored at −80 °C until sorting. Immature specimens were also collected from bamboo stems in St. Ann (Fig S2), discarded refrigerators in St. Elizabeth (Fig. 2e) and a discarded plastic container in Westmoreland using disposable plastic pipettes. Larvae were reared to adults in site-collected water under standard laboratory conditions. All mosquitoes were morphologically identified using a Leica S9E stereomicroscope (Leica, Germany) and taxonomic keys to the species level [37, 38]. Select specimens in exceptional morphological condition were randomly selected, stored in tubes containing silica, and shipped to Johns Hopkins Bloomberg School of Public Health for molecular analysis.

### DNA extraction, sequencing, mitogenome assembly and annotation

A modified extraction protocol was used to process single mosquito specimens which involved homogenization in a mixture of 98 μL of PK buffer (Applied Biosystems, Waltham, MA) with 2 μL of Proteinase K (Applied Biosystems, Waltham, MA) and incubation at 56 °C as previously described [39, 40]. Following the manufacturer’s instructions (Qiagen DNeasy Blood and Tissue Kit, Hilden, Germany), DNA was extracted from the homogenate and quantified using the Qubit dsDNA assay kit (Thermo Fisher Scientific, Waltham, MA) prior to library construction and Illumina sequencing at the SeqCenter (Pittsburgh, USA). Using a genome skimming strategy, libraries were sequenced (2 × 150) to a depth of 13.3 million reads. Using the reference, *Ae*. *aegypti* (NC_035159.1) mitochondrial genome as the seed sequence and kmer set at 39, the *Ae*. *vittatus* mitogenomes were assembled in NOVOPlasty (RRID:SCR_017335) version 4.3.5 [41]. Automatic annotations using the invertebrate genetic code under default settings were identified in MITOchondrial genome annotation [42] on the publicly available Galaxy EU platform [43]. To match reference *Aedes* in the GenBank repository, start and stop codons were manually adjusted in Geneious Prime (RRID:SCR_010519) version 2025.0.3 (Biomatters, Auckland, New Zealand). Overall, two complete annotated mitochondrial genomes representing *Ae*. *vittatus* were submitted to GenBank.

### Phylogenetic analysis

Short COI gene sequences were used for phylogenetic inference as there are no available complete mitogenomes of *Ae*. *vittatus* in GenBank. The COI gene sequences extracted from the mitogenomes generated in this study were aligned with available reference *Ae*. *vittatus* and *Ae*. *aegypti* COI sequences from GenBank using MAFFT implemented in Geneious Prime (RRID:SCR_010519) version 2025.0.3 (Biomatters, Auckland, New Zealand). The alignment file was exported in nexus formatted and imported into the jModelTest (v2.1.10) software to determine best fit pair substitution model [44]. Using Bayesian Evolutionary Analysis by Sampling Trees (BEAST) 2 [45], Bayesian inference analysis was performed using three independent runs under default settings: using a 20% burn in rate. FigTree v.1.4.4 was used to visualize trees (http://tree.bio.ed.ac.uk/software/figtree/).

## Results

### Collection of specimens

This is the first documented record of *Ae*. *vittatus* in Jamaica. Specimens were identified in collections sorted for viral metagenomic analysis from the parishes of St. Andrew, St. Ann, St. Elizabeth and Westmoreland (Table 1). Although collected in multiple locations in 2023 and 2024, *Ae*. *vittatus* mosquitoes were of relatively low abundance compared with most other species except at the St. Elizabeth and Westmoreland sites where they comprised at least one quarter of the collected mosquitoes. Upon inspection, several cut bamboos contained both water and larvae in St Ann (Fig S2), however, none of the larvae were *Ae*. *vittatus* (Table 1). Likewise, no *Ae*. *vittatus* larvae were found in the discarded container in Westmoreland (Table 1). Abandoned refrigerators at the St. Elizabeth served as a breeding site where immature *Ae*. *vittatus* specimens were collected with *Ae*. *albopictus* larvae (Table 1).

**Table 1:** Mosquito species collected alongside *Aedes vittatus* in breeding and trap locations

Location	Collection date	Stage	Species (number)
Mona—location 1- St. Andrew	January, 2023	adult	*Culex quinquefasciatus* (120) *Culex nigripalpus* (20) *Aedes taeniorhynchus* (11) *Aedes* spp. (2) *Aedes aegypti* (2) *Aedes albopictus* (1) *Aedes vittatus* (4) *Anopheles* spp. (1) *Aedes taeniorhynchus* (1) *Aedes vittatus* (1) *Culex quinquefasciatus* (149) *Culex nigripalpus* (15) *Culex nigripalpus* (105) *Culex quinquefasciatus* (880) *Aedes aegypti* (5) *Aedes vittatus* (4)
	February, 2023	adult	
	May, 2023	adult	
Mona—location 2- St. Andrew	February, 2023	adult	*Culex nigripalpus* (118) *Culex quinquefasciatus* (46) *Aedes* spp. (1) *Aedes vittatus* (2)
Mona—location 3- St. Andrew	July, 2024	adult	*Aedes albopictus* (10) *Aedes walkeri* (1) *Aedes aegypti* (11) *Culex nigripalpus* (4) *Aedes taeniorhynchus* (16) *Culex quinquefasciatus* (8) *Aedes vittatus* (2)
Walkerswood—St. Ann	October, 2023	adult	*Aedes albopictus* (60) *Aedes walkeri* (36) *Aedes aegypti* (1) *Aedes inaequalis* (37) *Wyeomyia* spp. (12) *Aedes vittatus* (4) *Culex nigripalpus* (2) *Aedes albopictus* (10) *Aedes inaequalis* (7)
		larvae	
Myersville—St. Elizabeth	September, 2024	adult	*Aedes aegypti* (1) *Aedes vittatus* (3) *Aedes albopictus* (30) *Aedes vittatus* (24)
		larvae	
Petersfield—Westmoreland	June, 2024	adult	*Aedes albopictus (9)* *Aedes mediovittatus (19)* *Aedes taeniorhynchus (2)* *Culex* spp. (7) *Aedes vittatus* (13) *Aedes albopictus* (3)
		larvae	

### Morphological and Molecular Identification of *Ae*. *vittatus* mosquitoes

**Fig. 3:**
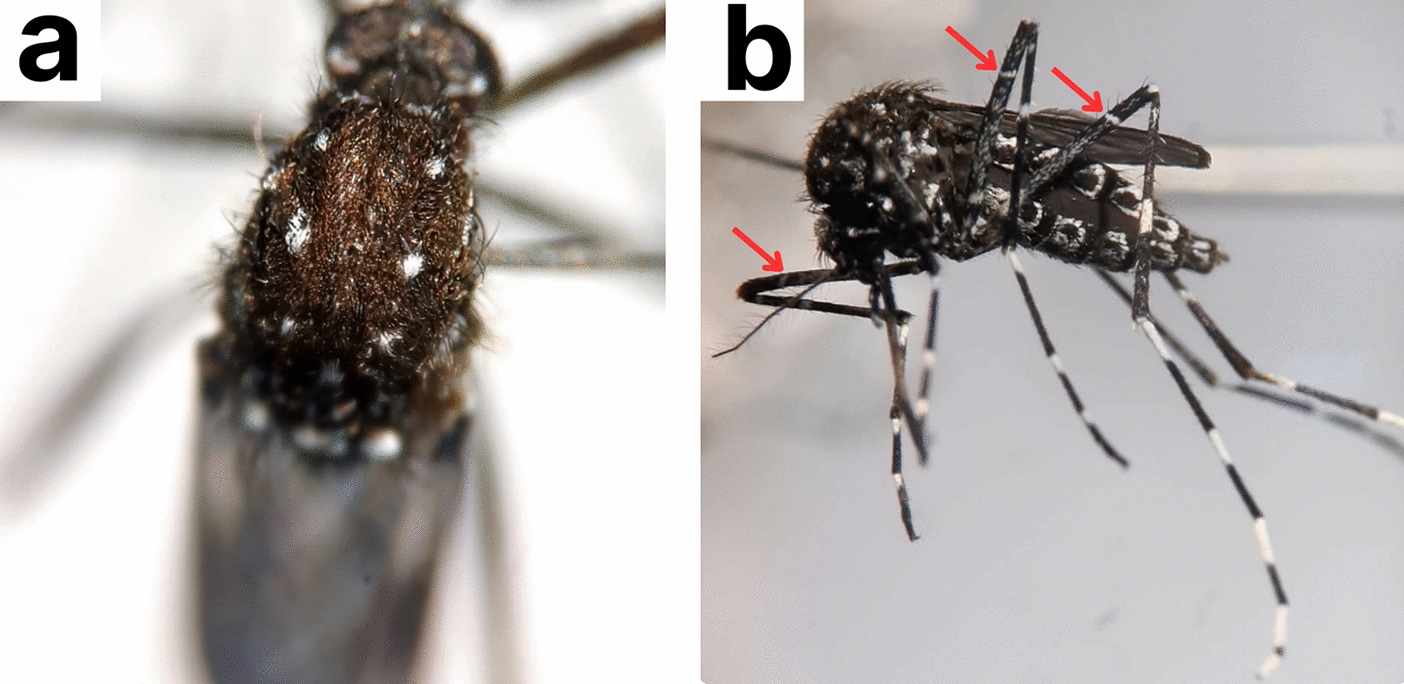
Morphological features of *Ae*. *vittatus*. **a** Scutum showing three pairs of slivery white spots. **b** All tibia with sub-basal white band

Morphological identification of *Ae*. *vittatus* was confirmed by the presence of three pairs of small, spherical, silvery white spots on its scutum and white band at the base of the tibiae as seen in (Fig. 3), using a taxonomic key provided by the Walter Reed Biosystematics Unit [38].

**Fig. 4:**
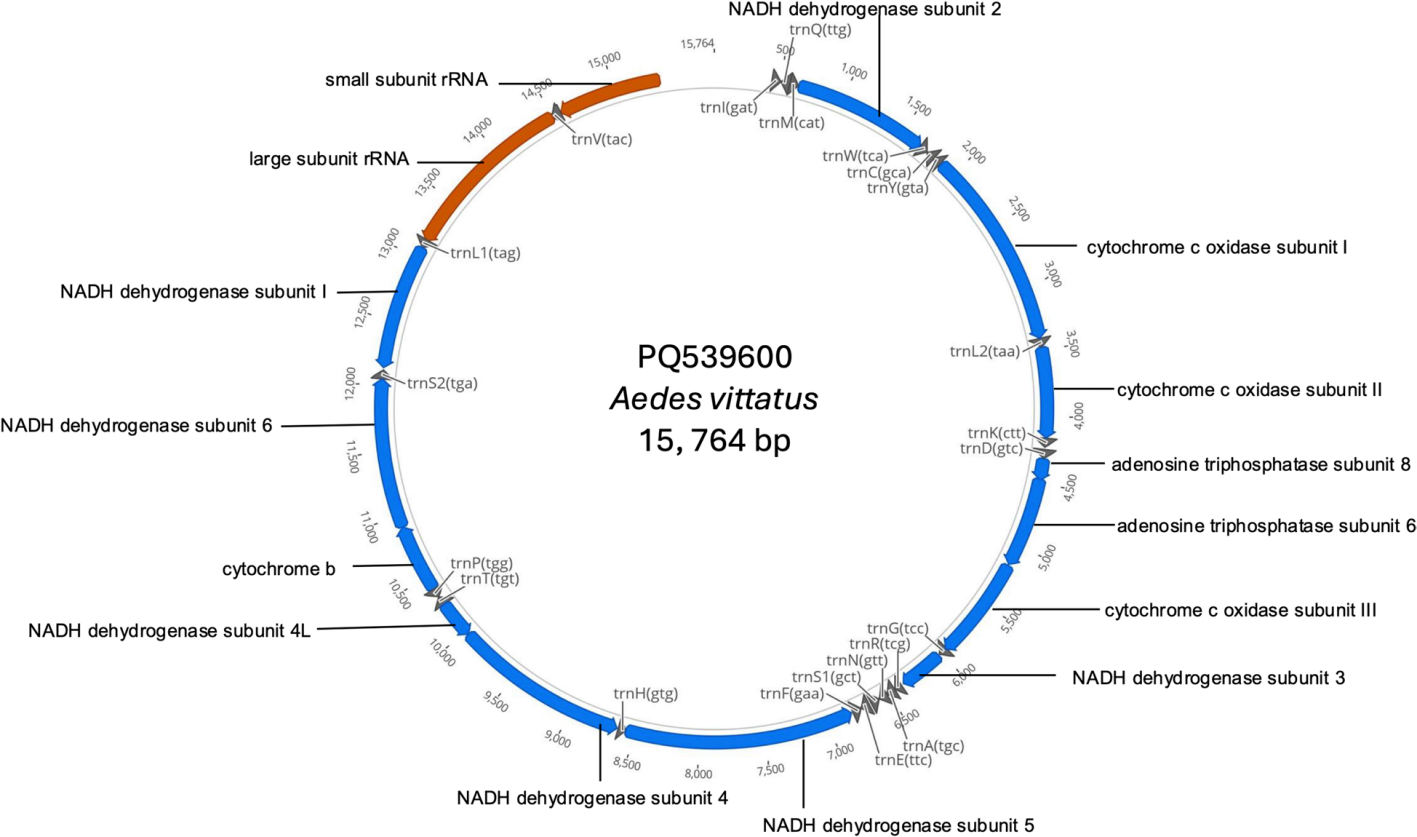
Representative mitochondrial genome map of *Ae*. *vittatus* from Mona, location 3 Jamaica. The blue, gray and red color blocks represent protein coding genes, transfer RNAs and ribosomal RNAs respectively

To link morphological and molecular identification of *Ae. vittatus,* two high quality morphological specimens were randomly selected for Illumina sequencing and mitochondrial genome assembly. The mitogenomes generated in this study were 15,764 and 15,785 bp in length, with an average AT content of 79.3%. *Aedes vittatus* mitogenomes were comparable with other *Aedes* species with 37 genes: 13 protein coding genes, 22 transfer RNAs, and 2 ribosomal RNA. The representative mitogenome map with annotated genes of *Ae*. *vittatus* is shown in (Fig. 4).

**Fig. 5:**
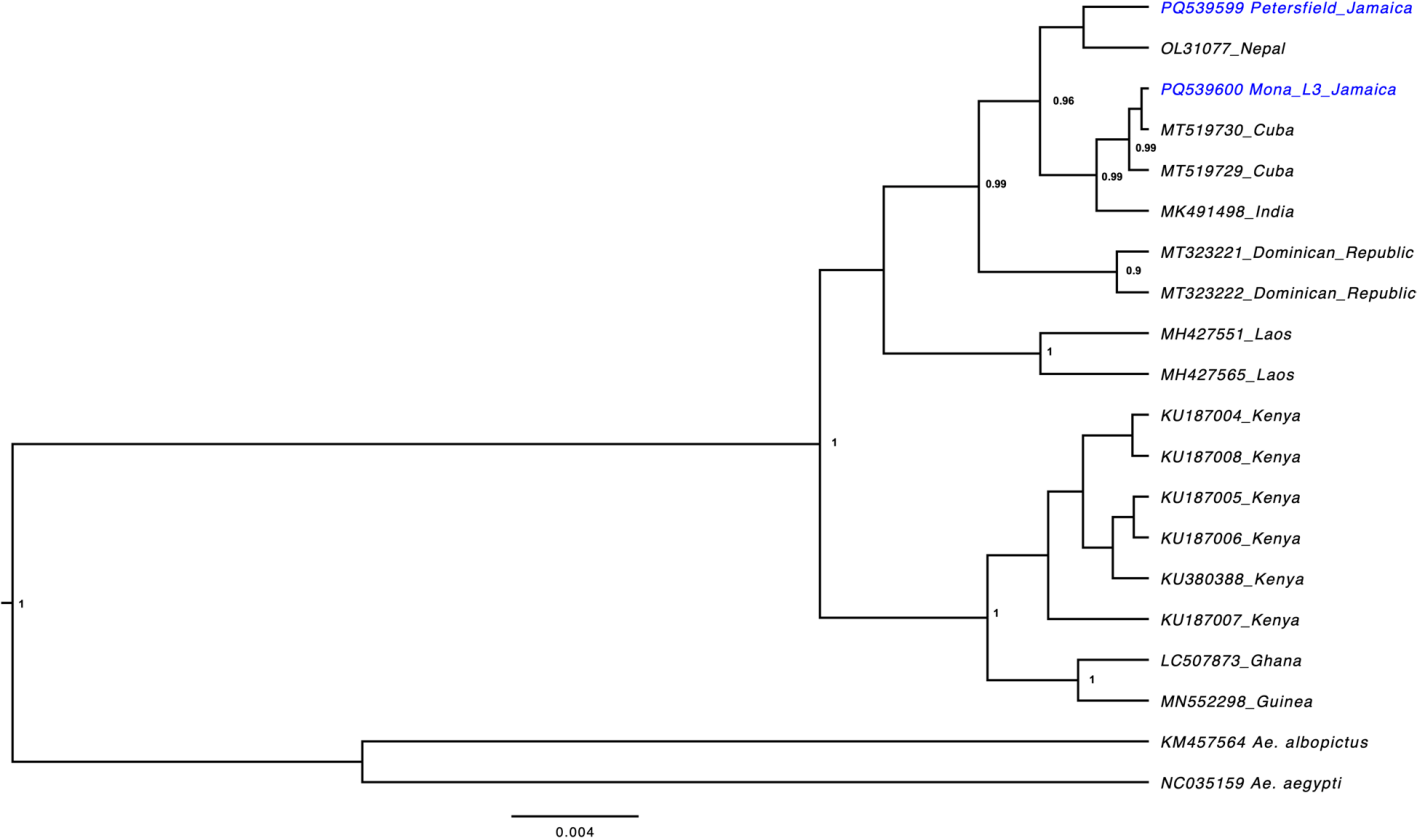
Phylogenetic tree for COI gene extracted from the mitogenomes generated in this study and available reference *Ae*. *vittatus* sequences. The numbers at the nodes represent posterior probabilities based on Bayesian inferences. The tree was constructed using BEAST 2 with the Tamura 3-parameter model

Since no other *Ae*. *vittatus* mitogenome exists in the public domain, the COI gene segment (1531 bp in length) was extracted from each mitogenome and added to an alignment matrix (539 bp) with 15 *Ae*. *vittatus* COI sequences available from the GenBank repository. Results from the 539 bp COI fragment BLAST analysis showed that PQ539599 from Petersfield shared 99.63% identity with OL331077, which was obtained from a dengue-endemic region of Nepal [46]. In contrast, PQ539600 from Mona, location 3 shared 100% identity with MT519730 from Cuba [14]. Bayesian inferences resulted in the Caribbean, Southeast Asia and Africa COI sequences separating into 3 main clades: with the Jamaican *Ae*. *vittatus* COI sequences clustering with sequences of *Ae*. *vittatus* collected in Cuba, India, and Nepal (Fig. 5).

## Discussion

The recent identification of invasive *Ae*. *vittatus* mosquito in Cuba, Dominican Republic and now in Jamaica is a significant finding with far-reaching implications for arbovirus transmission, surveillance, and vector control efforts on each island and the broader region of the Caribbean Sea. *Aedes vittatus* was identified in four parishes representing much of the geographic expanse of Jamaica. The extensive distribution of the species across the island suggests that it may have persisted undetected for some time but appears to be present in low densities, although the collections were relatively limited. The discovery of larvae indicates that *Ae*. *vittatus* populations are reproductively active and likely well-established.

*Aedes vittatus* an aggressive biter, is reported as a highly anthropophilic species, and was even suspected of playing a significant role in the initial transmission of yellow fever virus during the 1978–79 epidemic in The Gambia [47]. A laboratory infectivity study demonstrated *Ae*. *vittatus* competency in transmitting all four dengue serotypes [26]. Mavale et al. [26] indicated that although the infection rate of various dengue serotypes in *Ae*. *vittatus* was low, the mosquito salivary glands were infected, implying their potential to transmit or maintain the virus where dengue is endemic. A single study has also isolated dengue serotype 2 (DENV-2) from field-caught *Ae*. *vittatus* specimens in Senegal [48], and this species exhibited a higher dissemination rate of DENV-2 when compared with other vectors [49]. As the timing of the introduction of *Ae vittatus* into Jamaica is currently unknown, it raises the question about the potential role of this species in past arboviral epidemics. In the 2018–2019 dengue epidemic in Jamaica for instance, both dengue serotypes 2 and 3 were recorded among infected patients [50].

Moreover, it is worth noting that one of the locations where the *Ae*. *vittatus* was collected is a residential area of Mona. Although active breeding sites were not identified at this location, this indicates the species’ ability to adapt and coexist in peri-urban/urban environments. At Walkerswood there were signs of human activity, which is significant as human encroachment into forested habitats increases potential exposure to *Ae*. *vittatus* and the pathogens they may transmit. *Aedes vittatus* was also collected from Myersville, St Elizabeth, a rural farming community in Jamaica. The presence of discarded refrigerators which served as human-made breeding sites for *Ae*. *vittatus* in this forested area underscores the impact of humans in facilitating the introduction and establishment of new species. Although anthropophilic, studies have highlighted the potential of this vector to feed on both human and animals [51, 52] which can increase the possibility of acting as a bridge vector for zoonotic pathogens. Therefore, the identification of *Ae*. *vittatus* in rural and peri-urban communities throughout Jamaica needs to be further evaluated. To compound these concerns even further, is the scarcity of information on arboviral transmission regarding distribution of vectors, transmission competency and host use in Jamaica and the wider Caribbean. Hence, studies which seek to highlight and report the geographical and ecological expansion and adaptation of vectors and their competency for arboviruses, which are present in the region, are of paramount importance.

We have also reported the first complete mitogenomes of *Ae*. *vittatus* captured using a genome skimming approach. Sequencing of the entire mitogenome of *Ae*. *vittatus* provides more extensive gene coverage which can assist with more accurate molecular identification of this species and differentiation from other *Aedes* taxa. This is crucial for *Ae*. *vittatus* given the current misidentification of specimens as *Aedes cogilli* in GenBank which was highlighted in both the Cuban and Dominican Republic reports [13, 14]. The availability of only two mitogenomes for *Ae*. *vittatus*, both of which were generated from Jamaican samples has limited the analysis for this mosquito to date. Having a wider genomic dataset of *Ae*. *vittatus* mitogenomes from multiple locations throughout Jamaica and other geographically diverse areas will provide greater resolution in future phylogeographic studies. Additionally, incorporating additional genetic markers such as the nuclear Internal Transcribed Spacers 2 (ITS2) rDNA region as utilized in the Cuban study [14] can provide a complimentary source of resolution and diversify the genomic references currently available. Information obtained can provide invaluable insight into the population structure, introduction history and dispersal patterns of this invasive vector.

In this study both morphological identification and molecular verification were utilized to confirm the presence of *Ae*. *vittatus* in Jamaica. Phylogenetic analysis using a short 539 bp segment of the mitochondrial COI gene suggested multiple introductions of *Ae*. *vittatus* into Jamaica. Only a fragment of the mitochondrial COI gene was used as most genetic information available in the public domain for *Ae*. *vittatus* relates to this marker. Interestingly, the specimen from Mona, location 3 had 100% COI sequence identity to the Cuban sequence. This is suggestive of the potential role that increased trade partnerships can play in the introduction of invasive vectors. Further work will need to be undertaken to fully understand the origin(s) of the introduction of this mosquito species and dispersal throughout Jamaica.

It must be noted that convenience sampling limited the geographic coverage of our analysis and this report only included locations where *Ae*. *vittatus* was identified in the four parishes sampled between January 2023 and October 2024. Nevertheless, integration of these data into existing vector control programs could enhance their effectiveness and inform adjustments to current sampling strategies and interventions especially in rural areas. Future work will be expanded to surveil for *Ae*. *vittatus* at multiple locations in all parishes in Jamaica to better assess the distribution of *Ae*. *vittatus* on the island. Additionally, a phylogeographic approach will continue to be utilized to examine the population structure of *Ae*. *vittatus* to better understand its genetic history and introduction into Jamaica. These findings highlight the potential for new dynamics in vector-borne disease transmission and the need for more extensive vector surveillance on the island. Understanding the full extent of the current distribution of this vector in Jamaica will dictate the level of control that is required.

## Conclusions

This study presents the first confirmed detection of *Ae*. *vittatus* in Jamaica, highlighting a significant documentation of this invasive species into the island and the wider Caribbean. The generation of the first complete mitochondrial genome for *Ae*. *vittatus* provides a valuable genetic resource which can be used as a baseline for future vector surveillance and genomic assessment in the region. *Aedes vittatus* poses a growing public health threat due to its extensive geographic range and proven capacity to transmit various medically important arboviruses, such as yellow fever, dengue, chikungunya, and Zika viruses [12]. Furthermore, this species exhibits a propensity to reproduce in the vicinity of human habitats and has a strong attraction to seek out human hosts [11]. The detection of this vector in the Caribbean [13–15] raises concerns regarding its possible involvement in pathogen transmission, especially in areas where established vectors, such as *Ae*. *aegypti* and *Ae*. *albopictus*, are controlled. The species’ adaptation to diverse natural and human-made breeding sites increases its ability to survive and proliferate in new regions. Additionally, this species has demonstrated transovarial transmission of dengue virus, a powerful potential mechanism for sustaining viral circulation between outbreaks [53]. Owing to its invasive capability, enhanced by global trade and transportation, *Ae*. *vittatus* may pose an epidemiological threat in the Americas. Therefore, an increased and robust surveillance approach, grounded in regional communication and partnership can be used to promote awareness and the implementation of proactive control measures. These include prioritizing surveillance regions, activating early-warning systems, and facilitating resource distribution for focused control measures to prevent the potential introduction or spread of this invasive vector. Future work utilizing whole genome sequencing (WGS) of *Ae*. *vittatus* will be ideal for assessing genetic structure and gene flow dynamics, and may help to predict adaptive mutations that could propel distribution. It would also support epidemiological research to assess vector competence and arbovirus circulation. The present study lays the foundation for this future genomic work as well as comprehensive ecological investigations encompassing habitat use, seasonal dynamics, and possible range expansion across diverse Caribbean habitats for this new and emerging vector.

## Supplementary Information

Additional file 1.

Additional file 2.

## Data Availability

The dataset generated in this study is available on the NCBI database BioProject accession PRJNA1172362. The assembled mitochondrial sequences are openly available in the NCBI GenBank database under the accession numbers PQ539599–PQ539600.

## Abbreviations

*Ae*.: *Aedes*
COI: Cytochrome c oxidase subunit I
DENV-2: Dengue serotype 2
ITS2: Internal Transcribed Spacers 2
WGS: Whole genome sequencing
