# Supplementary figures and images for: Detection of invasive Aedes vittatus mosquitoes in Jamaica: molecular identification and surveillance implications
Source: Parasit Vectors. 2025 Nov 18;18:469. doi: 10.1186/s13071-025-07066-6 (PMC12625510; doi:10.1186/s13071-025-07066-6)

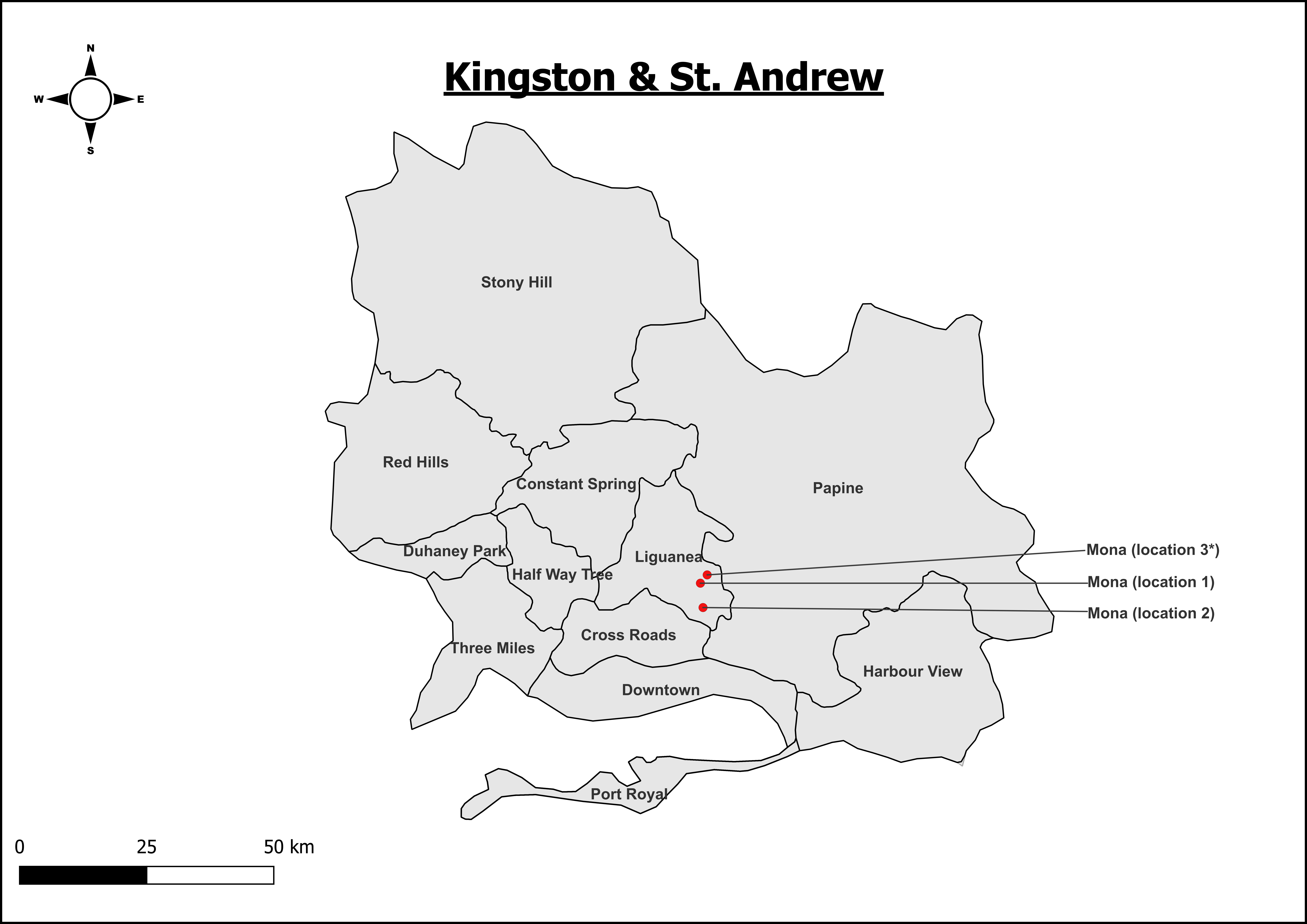

Supplement: Supplementary file 1 — Additional file 1. [file 13071_2025_7066_MOESM1_ESM.tif]

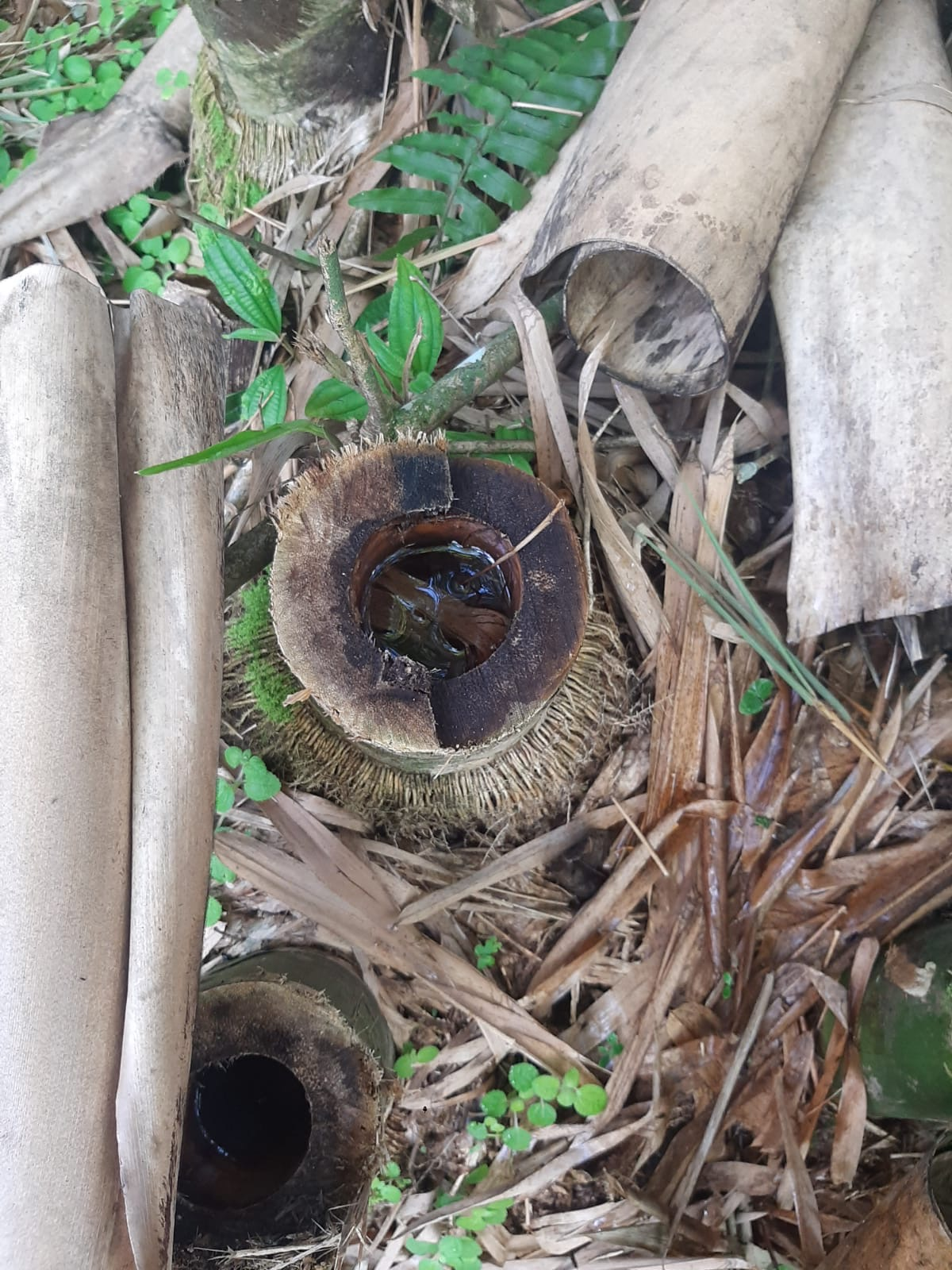

Supplement: Supplementary file 2 — Additional file 2. [file 13071_2025_7066_MOESM2_ESM.tiff]
